# Evidence for safety of retreatment with a single intra-articular injection of Gel-200 for treatment of osteoarthritis of the knee from the double-blind pivotal and open-label retreatment clinical trials

**DOI:** 10.1186/s12891-016-1101-0

**Published:** 2016-06-01

**Authors:** Vibeke Strand, Sooyeol Lim, Junko Takamura

**Affiliations:** Division of Immunology and Rheumatology, Stanford University School of Medicine, Palo Alto, CA USA; North American Business Unit, Seikagaku Corporation, Chiyoda-ku, Tokyo, Japan; Clinical Development Department, Research & Development Division, Seikagaku Corporation, Chiyoda-ku, Tokyo, Japan

**Keywords:** Osteoarthritis, Knee, Viscosupplementation, Intra-articular, Hyaluronic acid, Cross-linked HA, Gel-200

## Abstract

**Background:**

Gel-200 is a cross-linked hyaluronate single-injection device for treatment of osteoarthritis pain in the knee. This report summarizes new analyses of the safety of retreatment with Gel-200 from the 13-week, pivotal, multicenter, randomized controlled trial (RCT) followed by an open-label extension trial (OLE).

**Methods:**

379 patients were enrolled in the RCT [Gel-200; phosphate-buffered saline (PBS)]. Safety of retreatment with Gel-200 was assessed by comparing adverse events (AEs) and device-related AEs reported through Week 4 following retreatment with Gel-200 to those reported in patients receiving their first injection in the OLE.

**Results:**

350 patients completed the initial RCT (231 Gel-200; 119 PBS); 258 patients enrolled in the OLE (162 Gel-200; 96 PBS). In total, 202 patients (125 Gel-200; 77 PBS) qualified for retreatment, while 56 (37 Gel-200; 19 PBS) did not. There were no significant demographic or disease characteristic differences between Gel-200 patients who were and were not retreated; those who were not eligible for retreatment experienced greater pain relief from Gel-200 in the RCT by all effectiveness endpoints (all *p* < 0.001), without differences in their safety profile. In the OLE, the safety of Gel-200, including percentages of patients who experienced any AEs (*p* = 0.547) and device-related AEs (*p* = 0.521), did not significantly differ between those receiving a second versus a first injection of Gel-200 following PBS in the RCT.

**Conclusion:**

In the OLE, the safety of a second injection of Gel-200 was comparable to that of a first injection and effectiveness was similar, as previously reported.

**Trial registration:**

ClinicalTrials.gov identification numbers NTC 00449696 and NTC 00450112

## Background

Osteoarthritis (OA) is the most common inflammatory joint disorder in the world and one of the leading causes of disability [1]. Intra-articular (IA) injection of hyaluronic acid (HA) which is a glycosaminoglycan chain that occurs naturally within the knee joint, or viscosupplementation, is a widely used treatment for relief of OA pain in the knee [2, 3]. In addition to the mechanical effect of HA lubricating the joint and protecting the cartilage from mechanical degradation, IA-HA treatment is believed to exert its therapeutic effect by providing chondroprotection and anti-inflammatory effect, stimulating endogenous proteoglycan and HA synthesis, limiting subchondral bone changes, and reducing the action of joint nociceptors [4, 5]. However, recent OA treatment guidelines by American Academy of Orthopaedic Surgeons (AAOS) and American College of Rheumatology (ACR) cast doubt on the utility of this therapy [6, 7]. This stands in contrast to the fact that many clinicians have found HA to be a valuable treatment option for those OA patients with cardiovascular, gastrointestinal, and metabolic comorbidities which render use of many systemic drugs unsuitable [8]. Further analysis of clinical trial data should provide clinicians with more clarity on the utility of IA - HA over the discrepancy between guidelines and clinical practice.

Gel-200 (Gel-One^®^, Seikagaku Corporation, Tokyo, Japan) is a sterile, transparent, and viscoelastic hydrogel composed of a cross-linked hyaluronate, a derivative of a highly purified hyaluronate product. It was approved by the United States (US) Food and Drug Administration (FDA) in 2011 for the treatment of OA pain in the knee. The safety and effectiveness of a single Gel-200 injection was demonstrated in a 13-week pivotal multicenter randomized controlled trial (RCT) conducted in the US that recruited 379 OA patients. Analyses of the Western Ontario and McMaster Universities Osteoarthritis Index (WOMAC) visual analog scale (VAS) pain and physical function subscores, Outcome Measures in Rheumatology Clinical Trials and Osteoarthritis Research Society International (OMERACT − OARSI) strict responders, and physician global assessments of disease activity in the initial RCT demonstrated statistically significant improvements in patients treated with a single injection of Gel-200 compared with a phosphate-buffered saline (PBS) control. At 13 weeks, 6.39 mm of treatment difference demonstrated a statistical significance in a 100-mm VAS WOMAC pain subscore (*p* = 0.037), and from 3 weeks through 13 weeks, 7.10 mm of treatment difference was shown (*p* = 0.005). Improvements in WOMAC pain subscore were evident as early as 3 weeks following injection with more than 40 % improvement from baseline and continued through 13 weeks. Adverse events (AEs) were not significantly different between the treatment groups, and no unanticipated treatment-related serious AEs were reported [9].

Patients who completed the 13-week RCT were eligible to enroll in the open-label extension (OLE). Patients were eligible for treatment with Gel-200 at any point while enrolled in the extension once they met the eligibility criteria. Retreatment with Gel-200 was shown to be safe for 13 weeks post retreatment by comparing AE data between treatment groups (retreatment with Gel-200 and initial treatment with Gel-200 following PBS), although the previous analysis did not provide in-depth examination of comparability between the two groups. In addition, a previously published analysis demonstrated that repeat treatment with Gel-200 for 13 weeks following retreatment relieved symptomatic OA as effectively as the initial injection with a favorable safety profile [10]. For 13 weeks post retreatment, more than 30 mm improvement from baseline in WOMAC pain subscores persisted. The amount of pain relief provided by retreatment was greater than that reported over 13 weeks post initial treatment.

We report the results of additional analyses of the RCT and OLE data conducted to provide further evidence of the safety of a repeat injection of Gel-200.

## Methods

### Trial design

Both the RCT, SI-6606/01, and the OLE, Gel/1132, were approved by a central institutional review board. The RCT was conducted from August 2006 to December 2007 (last patient visit) at 28 sites in the US, and the OLE was conducted from March 2007 to May 2008 (last patient visit) at 23 of those sites in accordance with good clinical practices. A signed consent form approved by the institutional review board was obtained for each patient. These trials were registered with ClinicalTrials.gov (identification numbers NTC 00449696 and NTC 00450112, respectively). Patients were eligible for enrolment in the 13 week RCT if they had pain >4 weeks, Kellgren-Lawrence grade 1–3 by X-ray, and WOMAC pain subscore ≥40 mm. Patients were instructed to use only those medications during the trial for OA they were receiving prior to enrolment, as well as not to use any medications for symptomatic pain relief within the 24 h prior to each evaluation visit. Patients were randomized to Gel-200 or PBS in 2:1 ratio and followed up to 13 weeks.

All patients who completed the 13-week RCT were eligible to enroll in OLE following an informed consent to the extension and retreatment study. Patients who enrolled in the extension study did not receive a Gel-200 injection unless and until their WOMAC pain score again met eligibility for entry into the RCT, ie, ≥40 mm on VAS in the treated and ≤20 mm in the contralateral knee, at any time after enrollment or at evaluations at 3, 6, 9, and 13 weeks in the extension phase. After meeting these criteria, patients received a single injection of Gel-200 (30 mg cross-linked hyaluronate in 3.0 mL) in the treated knee at Week 0 of the retreatment phase and returned for evaluations at Weeks 1, 3, 6, 9, and 13 following reinjection. Patients who did not meet the pain criteria through Week 13 of the OLE did not receive another injection. All patients remained blinded to the initial treatment received during the RCT. Patients were again instructed to use only allowed medications and not initiate use of any new analgesic or anti-inflammatory agents during the study, as well as to not use any medications for symptomatic pain relief within the 24 h prior to each evaluation visit.

The safety and effectiveness in the 13-week RCT were assessed retrospectively by the population who received a second Gel-200 injection (Retreatment Group A) and who did not receive a second Gel-200 injection (Non-retreatment Group B)

The safety of retreatment with Gel-200 was assessed by comparing the rates of AEs and device-related AEs reported in the retreatment study through Week 4 in those patients receiving a second Gel-200 injection (Retreatment Group A) to the rates among patients receiving their first Gel-200 injection (ie, PBS patients from the RCT, PBS–Gel Group C).

### Statistical analyses

#### Analysis populations

**Fig. 1:**
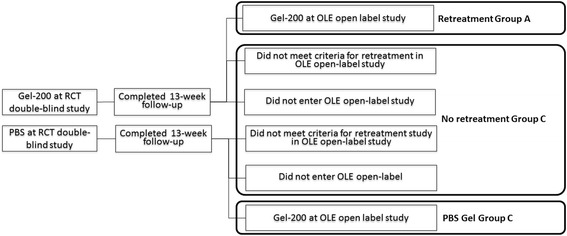
Composition of analysis populations

Data were analyzed by performing comparisons within 2 separate safety populations, diagramed in Fig. 1. Safety Population l comprised all patients who received Gel-200 treatment during the RCT study and were offered entry into OLE. This included 2 groups:

1. Retreatment Group A: patients who received treatment with Gel-200 in both the RCT and OLE
2. Nonretreatment Group B: patients who received treatment with Gel-200 in the RCT and either did not consent to or never met criteria for retreatment

Safety Population 2 comprised all patients who received a Gel-200 injection during the OLE. This population included 2 groups of patients:

1. Retreatment Group A: as defined above, patients who received treatment with Gel-200 in both the RCT and OLE
2. PBS–Gel Group C: patients who received PBS during the RCT and Gel-200 during the OLE

#### Statistical methods

To demonstrate the safety of retreatment with Gel-200, rates of AEs and device-related AEs up to 28 days after reinjection were compared between Retreatment Group A and PBS–Gel Group C using summary statistics and Fisher’s exact tests.

To assess the suitability of Retreatment Group A for assessment of the safety of retreatment with Gel-200, demographics and baseline disease characteristics, rates of AEs, rates of device-related AEs, and primary and major secondary efficacy endpoints in the initial RCT were compared between Retreatment Group A and Nonretreatment Group B using descriptive and summary statistics, Fisher’s exact tests, and t-tests, as appropriate.

## Results

### Patient population

**Fig. 2:**
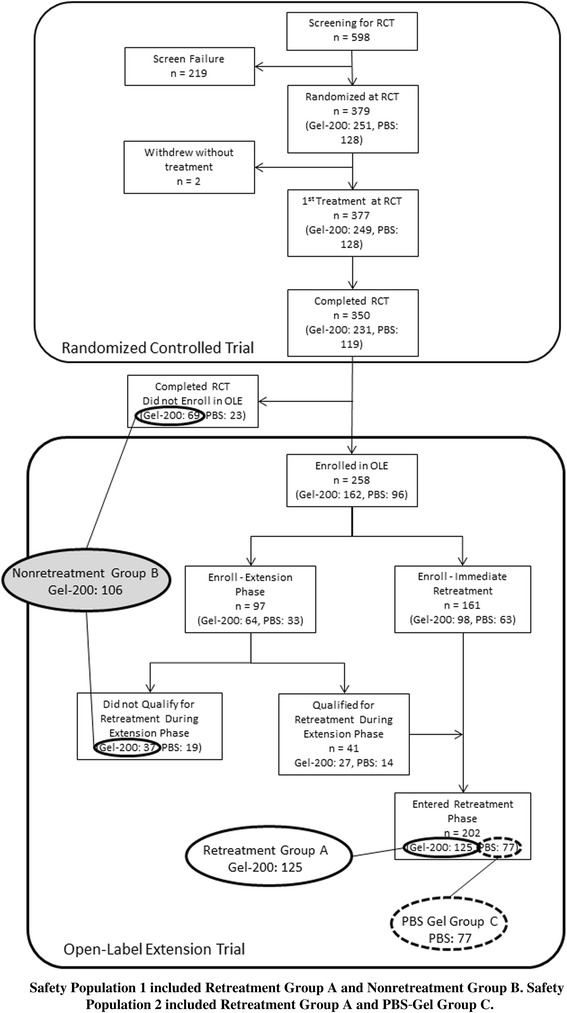
Flowchart of patient population, including identity of analysis populations

Of the 350 patients who completed the pivotal RCT and were eligible to enroll in the OLE, 258 patients (74 %) enrolled; 92 patients (26 %) did not, including 69 who received Gel-200 (Fig. 2). Of patients enrolled in the OLE, 162 (63 %) were previously treated with Gel-200 in the initial treatment study, 125 patients (48 %) received a second injection of Gel-200 during the OLE (Retreatment Group A). Of 96 patients who received PBS in the RCT, 77 (80 %) received a Gel-200 injection in the OLE. A total of 37 patients who were treated with Gel-200 in the RCT and enrolled in the OLE did not qualify for retreatment and were combined with the 69 patients who received Gel-200 in the RCT but did not enroll in the OLE, for a total of 106 in Nontreatment Group B.

### Demographics of retreatment group A versus nonretreatment group B during the initial treatment study

There were no significant demographic or baseline differences between Retreatment Group A and Nonretreatment Group B (Table 1). The mean age of patients was approximately 61 years, and approximately 59 % were female in both groups. The overall physical profile of patients in both groups was similar, including mean BMI of 28.4 kg/m^2^ and 28.3 kg/m^2^, respectively. The most common Kellgren-Lawrence score in both groups was 3, reported by 46.2 % of patients in Nonretreatment Group B and 58.4 % of patients in Retreatment Group A. The mean baseline of WOMAC pain subscores in the study knee were 68.1 and 69.0, respectively.

**Table 1:** Patient Demographics in Retreatment Group A and Nonretreatment Group B

	Nonretreatment Group B	Retreatment Group A
	(*N* = 106)	(*N* = 125)
Age, years		
Mean (SD)	61.4 (10.00)	60.8 (10.39)
Gender, N (%)		
Female	62 (58.5)	74 (59.2)
Male	44 (41.5)	51 (40.8)
Body mass index, kg/m^2^ (mean ± SD)	28.4 (4.06)	28.3 (4.05)
Kellgren-Lawrence Score - Study Knee [N (%)]		
Grade 1	11 (10.4)	10 (8.0)
Grade 2	46 (43.4)	42 (33.6)
Grade 3	49 (46.2)	73 (58.4)
WOMAC pain subscores - Study Knee (mean)	68.1	69.0

No statistically significant difference was identified between groups

### Safety during the initial treatment study in retreatment group A versus nonretreatment group B

The percentages of patients who experienced AEs and device related AEs in the pivotal RCT were similar between Retreatment Group A and Nonretreatment Group B without statistically significant difference (Table 2). The most common AEs in both groups, reported by ≥5 % of patients, included joint swelling, joint effusion, arthralgia, and upper respiratory tract infection (URI). Rates were similar between the 2 groups for all of these AEs except for URIs, reported by 10.4 % in Retreatment Group A and 2.8 % in Nonretreatment Group B. Most common related AEs reported by ≥5 % of patients were joint swelling, joint effusion, and arthralgia. Rates of specific related AEs were also similar between the 2 treatment groups. Overall, the profile of the safety data collected in the RCT was similar between patients who did and did not receive retreatment with Gel-200 in the OLE.

**Table 2:** Overall Summary of Adverse Events in Retreatment Group A and Nonretreatment Group B

	Nonretreatment Group B		Retreatment Group A	
	(*N* = 106)		(*N* = 125)	
	Events	Patients (%)	Events	Patients (%)
AEs	187	73 (68.9)	233	84 (67.2)
AEs occurring in ≥5 % of patients				
Joint swelling	33	27 (25.5)	49	37 (29.6)
Joint effusion	29	24 (22.6)	35	30 (24.0)
Arthralgia	22	17 (16.0)	24	19 (15.2)
Upper respiratory tract infection	3	3 (2.8)	13	13 (10.4)
Related AEs	48	27 (25.5)	59	31 (24.8)
Related AEs occurring in ≥5 % of patients				
Joint swelling	15	14 (13.2)	22	15 (12.0)
Joint effusion	13	12 (11.3)	14	12 (9.6)
Arthralgia	9	7 (6.6)	13	10 (8.0)

No statistically significant difference was identified between groups

### Effectiveness during the initial treatment study in retreatment group A versus nonretreatment group B

For all assessments at all timepoints following initial treatment in the pivotal RCT (Weeks 3, 6, 9, and 13), there was a statistically significant difference between baseline and follow-up VAS WOMAC pain subscores within each group (all *p* <0.001). Mean scores and changes from baseline were also compared between the 2 groups. For all measures at all timepoints following treatment, mean scores and changes from baseline were significantly different between analysis groups (Weeks 3, 6, and 9 for the change from baseline in WOMAC stiffness subscores, *p* = 0.002; all others, *p* <0.001). At Week 13, the endpoint in the initial RCT, there were statistically significant differences (all *p* <0.001) between mean results in the 2 analysis groups for all WOMAC subscores, patient and physician global evaluation (Table 3). In all cases, mean changes from baseline were larger in Nonretreatment Group B compared with Retreatment Group A, indicating that patients experienced greater OA pain relief in Nonretreatment Group B.

**Table 3:** Summary of Effectiveness of Gel-200 at Week 13 in Retreatment Group A and Nonretreatment Group B

Outcome	Nonretreatment Group B versus Retreatment Group A	
	Difference (95 % CI)	*p*-value
WOMAC pain subscores	26.1 (19.2, 32.9)	<0.001
WOMAC physical function subscores	23.3 (16.4, 30.2)	<0.001
WOMAC stiffness subscores	23.9 (16.6, 31.2)	<0.001
Total WOMAC score	23.9 (17.2, 30.6)	<0.001
Patient global evaluation	23.0 (14.9, 31.2)	<0.001
Physician global evaluation	17.6 (10.5, 24.7)	<0.001

### Safety of retreatment with Gel-200 in the retreatment study in retreatment group A versus PBS–Gel-200 group C

The percentages of patients who experienced AEs and device-related AEs through 28 days following treatment in the OLE were similar in Retreatment Group A and PBS–Gel Group C (*p* = 0.547 and *p* = 0.521, respectively; Table 4). The most commonly reported AEs were in the musculoskeletal and connective tissue disorders system organ class, with the most common preferred terms, reported by ≥5 % of patients, being joint effusion, arthralgia, and joint swelling. Rates of specific device-related AEs were also similar between the 2 treatment groups. Overall, the safety profile following treatment with Gel-200 in the OLE was similar between patients receiving a second Gel-200 injection (retreatment) and those receiving an initial injection after receiving PBS in the pivotal RCT.

**Table 4:** Overall Summary of Adverse Events in Retreatment Group A and PBS Gel Group C

	PBS Gel–Group C		Retreatment Group A	
	(*N* = 74)		(*N* = 125)	
	Events	Patients (%)	Events	Patients (%)
AEs	38	26 (35.1)	76	50 (40.0)
TEAEs occurring in ≥5 % of patients				
Joint effusion	7	7 (9.5)	13	13 (10.4)
Arthralgia	6	6 (8.1)	12	12 (9.6)
Joint swelling	7	7 (9.5)	10	10 (8.0)
Related AEs	12	8 (10.8)	31	18 (14.4)
Related AEs occurring in ≥5 % of patients				
Arthralgia	5	5 (6.8)	9	9 (7.2)
Joint swelling	3	3 (4.1)	7	7 (5.6)

No statistically significant difference was identified between groups

## Discussion

A recent meta-analysis of 29 studies of FDA-approved viscosupplements by Strand et al. demonstrated that these products are both safe and effective through 26 weeks in patients with symptomatic knee OA, which included the pivotal RCT of single injection Gel-200 that demonstrated its safety 13 weeks post injection [11]. Consistent with the findings of this meta-analysis, a recent consensus statement issued by experts in Europe also confirmed the safety and effectiveness of viscosupplementation in clinical practice [12], in contrast to consensus statements in the US by AAOS and Osteoarthritis Research Society International (OARSI) [6, 13]. However, reports in the literature regarding the safety and effectiveness of viscosupplementation in patients with knee OA have been mixed. In a comprehensive meta-analysis of 89 trials that compared viscosupplementation with sham or a nonintervention control in adults with knee OA, Rutjes et al. reported that viscosupplementation was associated with a small, clinically irrelevant benefit and an increased risk of serious AEs and local AEs; however, the authors also noted that trial quality was generally low and safety data were often poorly reported [14]. Another publication countered that viscosupplementation is safe [8, 15]. Thus, safety data continue to be analyzed for viscosupplementation products, and this issue has become more germane as safety of alternative treatments for knee OA is being debated [16].

Multiple single-injection viscosupplementation products are approved in the US for which the safety of a repeat injection has been examined [17–19]. For two of these products, follow-up occurred for at least 4 weeks after the reinjection, and 4 weeks of safety data were used as evidence for safety of a repeat injection. Thus, although a previously published analysis of the OLE study had demonstrated safety of a repeat injection of Gel-200 up to 13 weeks following retreatment [10], the current analysis examined safety data over 4 weeks following reinjection. The incidences of all AEs and device-related AEs were similar between patients receiving retreatment with Gel-200 and those receiving an initial injection. These results, consistent with the meta-analysis by Strand et al., indicate that retreatment with Gel-200 is generally safe and well tolerated [11]. Additionally, when comparing reported AE rates of a repeat injection of Gel-200 over 13 weeks, AE rates at 4 weeks were much lower than those over 13 weeks [10]. No acute AE that may raise a concern on safety of Gel-200 was identified.

The retreatment population rate in this study was lower than those reported in retreatment studies for similar products. Fewer patients who completed the pivotal Gel-200 RCT received retreatment in the Gel/1132 study. For example, in trials for approved viscosupplementation products Synvisc-One^®^, MONOVISC^®^, and EUFLEXXA^®^, reinjection rates of 63, 65, and 74 %, respectively, were reported, compared with a 53 % reinjection rate for Gel-200 in the OLE [17–19]. It was therefore important to determine that the Retreatment Group A was appropriate to include in this safety evaluation, representing the overall Gel-200 treatment population in the pivotal RCT. Overall, the patients retreated with Gel-200 in the OLE were similar to those who were not reinjected. There were no significant differences in demographics, baseline characteristics, or safety events reported in the pivotal RCT study for those who did and did not receive a second Gel-200 injection in the following OLE. These results show that the Retreatment Group A was an appropriate sample with which to examine the safety of retreatment with Gel-200. Moveover, patients who were not reinjected also did not appear to have any specific safety concerns about Gel-200 that would have precluded reinjection. Upper respiratory infections were reported by ≥5 % in patients with Gel-One in the 13-week RCT, also in patients receiving PBS, with a similar incidence (4.7 %). In both treatment groups, all cases were judged as not related to treatment by investigators. Nonretreatment patients also did not appear to have any concerns regarding effectiveness; patients in Nonretreatment Group B experienced greater OA pain relief following Gel-200 during the RCT than patients in Retreatment Group A. Additionally, those patients who did not consent to enter the OLE had better effectiveness results at Week 13 in the pivotal study. The high pain-relieving effect experienced by patients who received Gel-200 injection in Nonretreatment Group B may have contributed to their decision not to enter the retreatment study (data not shown), as it is plausible that for many of these patients, no retreatment was needed at the time that it was provided in the OLE study. Such potential evidence of long-lasting effectiveness of Gel-200 is consistent with the reporting of a recent network meta-analysis by Bannuru et al., which showed that HA injections were more effective in pain relief than other pharmacologic interventions once the effect of PBS injection serving as an active control was accounted for during the network meta-analysis [20, 21]. In the case of Gel-200, the potential evidence for long-lasting analgesic effect of Gel-200 in the RCT is corroborated by the finding from a previous animal study which showed that Gel-200 remained in the joint even 28 days after injection [22]. There was no specific feature in patients who received the second Gel-200 injection. Patient demographic between patients who received the second Gel-200 injection and those who did not receive the second Gel-200 injection, were similar except for pain scores. However, these results also demonstrate internal consistency with previous findings from the same trial that those receiving an initial injection of Gel-200 had a longer time before meeting the threshold condition for retreatment eligibility than patients receiving PBS [10].

## Conclusions

In conclusion, these additional analyses of retreatment from the OLE have confirmed the safety of Gel-200 following single and repeat injections in both pivotal and retreatment trials. Acute AEs were much lower and similar in incidence to non-acute AEs.

Moreover, pain scores before the retreatment indicated that non-retreated patients reported more improvement in pain than patients who ultimately received retreatment, suggesting that Gel-200 was sufficiently effective to obviate the need for retreatment in many patients in the RCT.

## Abbreviations

AAOS, American Academy of Orthopaedic Surgeons; ACR, American College of Rheumatology; AE, adverse event; FDA, Food and Drug Administration; HA, hyaluronic acid; IA, intra articular; OA, osteoarthritis; OARSI, Osteoarthritis Research Society International; OLE, open-label extension trial; OMERACT − OARSI, Outcome Measures in Rheumatology Clinical Trials and Osteoarthritis Research Society International; PBS, phosphate-buffered saline; RCT, pivotal, multicenter, randomized controlled trial; US, United States; VAS, visual analogue scale; WOMAC, Western Ontario and McMaster Universities Osteoarthritis Index

## References

[1] Buckwalter JA, Saltzman C, Brown T (2004). The impact of osteoarthritis: implications for research. Clin Orthop Relat Res.

[2] Goldberg VM, Goldberg L (2010). Intra-articular hyaluronans: the treatment of knee pain in osteoarthritis. J Pain Res.

[3] Strand V, Conaghan PG, Lohmander LS, Koutsoukos AD, Hurley FL, Bird H (2006). An integrated analysis of five double-blind, randomized controlled trials evaluating the safety and efficacy of a hyaluronan product for intra-articular injection in osteoarthritis of the knee. Osteoarthritis Cartilage.

[4] Altman RD, Manjoo A, Fierlinger A, Niazi F, Nicholls M. The mechanism of action for hyaluronic acid treatment in the osteoarthritic knee: a systematic review. BMC Musculoskelet Disord. 2015;16:1–10. 10.1186/s12891-015-0775-z PMC4621876 26503103

[5] Migliore A, Procopio S (2015). Effectiveness and utility of hyaluronic acid in osteoarthritis. Clin Cases Miner Bone Metab.

[6] American Academy of Orthopaedic Surgeons. Treatment of osteoarthritis of the knee evidence-based guideline 2^nd^ edition. 2013. http://www.aaos.org/research/guidelines/TreatmentofOsteoarthritisoftheKneeGuideline.pdf. Accessed 22 September 2015.

[7] Hochberg MC, Altman RD, April KT, Benkhalti M, Guyatt G, McGowan J (2012). American college of rheumatology 2012 recommendations for the use of nonpharmacologic and pharmacologic therapies in osteoarthritis of the hand, hip, and knee. Arthritis Care Res.

[8] Migliore A, Bizzi E, Herrero-Beaumont J, Petrella RJ, Raman R, Chevalier X (2015). The discrepancy between recommendations and clinical practice for viscosupplementation in osteoarthritis: mind the gap!. Eur Rev Med Pharmacol Sci.

[9] Strand V, Baraf HSB, Lavin PT, Lim S, Hosokawa H (2012). A multicenter, randomized controlled trial comparing a single intra-articular injection of Gel-200, a new cross-linked formulation of hyaluronic acid, to phosphate buffered saline for treatment of osteoarthritis of the knee. Osteoarthritis Cartilage.

[10] Strand V, Baraf HSB, Lavin PT, Lim S, Hosokawa H (2012). Effectiveness and safety of a multicenter extension and retreatment trial of Gel-200 in patients with knee osteoarthritis. Cartilage.

[11] Strand V, Mclntyre LF, Each WR, Miller LE, Block JE (2015). Safety and efficacy of US approved viscosupplements for knee osteoarthritis: a systematic review and meta-analysis of randomized, saline-controlled trials. J Pain Res.

[12] Henrotin Y, Raman R, Richette P, Bard H, Jerosch J, Conrozier T (2015). Consensus statement on viscosupplementation with hyaluronic acid for the management of osteoarthritis. Semin Arthritis Rheum.

[13] Zhang W, Moskowitz RW, Nuki G, Abramson S, Altman RD, Arden N (2008). OARSI recommendations for the management of hip and knee osteoarthritis. Part II: OARSI evidence-based, expert consensus guidelines. Osteoarthritis Cartilage.

[14] Rutjes AW, Jüni P, Costa BR, Trelle S, Nüesch E, Reichenbach S (2012). Viscosupplementation for osteoarthritis of the knee: a systemic review and meta-analysis. Ann Intern Med.

[15] McAlindon TE, Bannuru RR (2012). Osteoarthritis: Is viscosupplementation really so unsafe for knee OA?. Nat Rev Rheumatol.

[16] Lu N, Misra D, Neogi T, Choi HK, Zhang Y (2015). Total joint arthroplasty and the risk of myocardial infarction — A general population propensity score-marched cohort study. Arthritis Rheum.

[17] Prescribing Information for Synvisc-One™ http://www.accessdata.fda.gov/cdrh_docs/pdf/p940015s012b.pdf.

[18] Prescribing Information for MONOVISC™ http://www.accessdata.fda.gov/cdrh_docs/pdf9/P090031b.pdf.

[19] Prescribing Information for EUFLEXXA® http://www.accessdata.fda.gov/cdrh_docs/pdf/P010029S008b.pdf.

[20] Bannuru RR, Natov NS, Obadan IE, Price LL, Schmid CH, McAlindon TE (2009). Therapeutic trajectory of hyaluronic acid versus corticosteroids in the treatment of knee osteoarthritis: a systematic review and meta-analysis. Arthritis Rheum.

[21] Bannuru RR, Schmid CH, Kent DM, Vaysbrot EE, Wong JB, McAlindon TE (2015). Comparative effectiveness of pharmacologic interventions for knee osteoarthritis: a systematic review and network meta-analysis. Ann Intern Med.

[22] Yoshioka K, Yasuda Y, Kisukeda T, Nodera R, Tanaka Y, Miyamoto K (2014). Pharmacological effects of novel cross-linked hyaluronate, Gel-200, in experimental animal models of osteoarthritis and human cell lines. Osteoarthritis Cartilage.

